# Detection of Antibodies against Turkey Astrovirus in Humans

**DOI:** 10.1371/journal.pone.0096934

**Published:** 2014-05-14

**Authors:** Victoria A. Meliopoulos, Ghazi Kayali, Andrew Burnham, Christine M. Oshansky, Paul G. Thomas, Gregory C. Gray, Melinda A. Beck, Stacey Schultz-Cherry

**Affiliations:** 1 Department of Infectious Diseases, St Jude Children’s Research Hospital, Memphis, Tennessee, United States of America; 2 Department of Immunology, St Jude Children’s Research Hospital, Memphis, Tennessee, United States of America; 3 Department of Environmental and Global Health, College of Public Health Professions, University of Florida, Gainesville, Florida, United States of America; 4 Department of Nutrition, Gillings School of Global Public Health, University of North Carolina at Chapel Hill, Chapel Hill, North Carolina, United States of America; Columbia University, United States of America

## Abstract

Astroviruses are a leading cause of gastroenteritis in mammals and birds worldwide. Although historically thought to be species-specific, increasing evidence suggests that astroviruses may cross species barriers. In this report, we used enzyme-linked immunosorbent assays to screen sera from three distinct human cohorts involved in influenza studies in Memphis, TN or Chapel Hill, NC, and Midwestern poultry abattoir workers for antibodies to turkey astrovirus type 2 (TAstV-2). Surprisingly, 26% of one cohort’s population was TAstV-2 positive as compared to 0 and 8.9% in the other cohorts. This cohort was composed of people with exposure to turkeys in the Midwestern United States including abattoir workers, turkey growers, and non-occupationally exposed participants. The odds of testing positive for antibodies against turkey astrovirus among abattoir workers were approximately 3 times higher than the other groups. These studies suggest that people with contact to turkeys can develop serological responses to turkey astrovirus. Further work is needed to determine if these exposures result in virus replication and/or clinical disease.

## Introduction

Astroviruses are a diverse family of small, non-enveloped RNA viruses that have been identified in a broad range of species, including humans, wild birds, poultry, bats, dogs, and marine mammals [1], [2], [3], [4], [5]. Surveillance using molecular diagnostic methods demonstrates that human astroviruses (HAstV) are one of the most important causes of pediatric acute gastroenteritis, although outbreaks affecting adults and the elderly are also frequently described [6]. Astroviruses can also be associated with asymptomatic infections and in the case of animal astroviruses, with non-enteric clinical diseases including hepatitis, nephritis, and neurologic complications [7].

The *Astroviridae* family is divided into distinct *Mamastrovirus* and *Avastrovirus* genera that share limited sequence identity. For example, the capsid proteins of turkey astrovirus-2 (TAstV-2) and human astrovirus type 1 (HAstV-1) share only 25% amino acid similarity and are structurally distinct [8]. Although astrovirus infections are thought to be species specific [7], the wide variety of species infected, the genetic diversity of the family, and the occurrence of recombination suggests that cross-species transmission of astroviruses could occur. In fact, distinct avian strains have been transmitted across bird species [9], [10], [11]. More recently, novel astrovirus strains have been discovered in humans that are genetically similar to animal viruses including rat and swine [12]. To date, there have been no known infections of mammals with avian astroviruses.

The objective of these studies was to screen human sera for antibodies against TAstV-2. Included in our cohorts were adults occupationally exposed to turkeys in the Midwestern US where TAstV-2 has been identified [11], [13]. Previous work has demonstrated that poultry meat packers and slaughterhouse workers have elevated antibody titers against a variety of avian viruses including avian metapneumoviruses [14], avian leukosis/sarcoma virus [15], reticuloendotheliosis virus [15], [16], and Marek’s disease virus [17]. Similarly we now report that this population also has TAstV-2 antibodies. Compared to other population samplings, we found a more robust and higher incidence of seroreactivity to TAstV-2. These studies provide evidence that avian astroviruses can induce serological responses in exposed humans but further studies are needed to determine if they are associated with virus replication and/or clinical disease.

## Materials and Methods

### Study Samples

Samples were collected in compliance with 45 CFR 46 and the Declaration of Helsinki. Institutional Review Boards of the University of Iowa, St Jude Children’s Research Hospital and the University of Tennessee Health Science Center/Le Bonheur Children’s Hospital approved the study. At the time of enrolment, written, informed consent was obtained from participants or their parents/guardians, and assent was obtained from age-appropriate participants. Limited quantities of archived human sera from cohorts involved in institutional review board (IRB) approved studies as described below were available for these studies. All samples were coded by the originating laboratories prior to providing them for astrovirus analysis.

Cohort A is an ongoing prospective study carried out at LeBonheur Children’s Hospital in Memphis, TN. Eligible participants included children and adults exhibiting influenza-like illness as described in [18], [19], [20]. Samples were collected from February to April 2012 in compliance with 45 CFR 46 and the Declaration of Helsinki. Institutional Review Boards of St Jude Children’s Research Hospital and the University of Tennessee Health Science Center/Le Bonheur Children’s Hospital approved the study.

Cohort B is an ongoing, prospective study carried out at the University of North Carolina Family Medicine Center, an academic outpatient primary care facility in Chapel Hill, NC as described in [21], [22]. Eligible participants were adults (greater than or equal to18 years) receiving the influenza vaccine and samples were collected between 2009 and 2011. All procedures were approved by the Biomedical Institutional Review Board at the University of North Carolina and St Jude Children’s Research Hospital.

Cohort C was an IRB-approved study at the University of Iowa. In the original cross-sectional study, from March 2007 to April 2008, a total of 57 turkey growers, 38 turkey meat processing plant workers, and 82 occupationally unexposed workers that did not interact with turkeys at the workplace on a daily basis from Iowa and Illinois were enrolled after written informed consent was obtained. Participants completed a questionnaire about their demographic, occupational, and general health status. A blood specimen for laboratory analysis was successfully obtained from 170 of 177 participants and serum was collected. The demographic characteristics of the study participants have been published [14]. 160 serum samples were available for this study.

### Capsid Protein Purification and Antibody Production

Recombinant HAstV-1 Oxford (GenBank L23513) and TAstV-2 (NC005790.1) astrovirus capsid proteins were expressed in baculovirus and purified as described [8], [23]. Protein concentration was measured by BCA assay (Pierce, Rockford, IL). HAstV-1 polyclonal antiserum was generated from rabbits immunized with HAstV-1 peptide (IPRSRASGHGYESD) and rabbit anti–TAstV-2 was generated from TAstV-2 peptide (KHLEEEKNYWKNQ) (ProSci, Poway, CA), epitopes within the acidic domain of the astrovirus capsid protein.

### Astrovirus Capsid ELISA

High binding–affinity polystyrene plates (Corning Incorporated, Corning, NY) were coated with 0.05 µg/well of the astrovirus capsid protein of interest or BSA (negative control) and incubated overnight at 4°C. Plates were washed thrice with PBS in 0.05% Tween-20 (PBST) and then blocked with 4% BSA in PBST for 2 hours at room temperature. Following extensive washing, diluted human sera samples (1∶100 unless otherwise noted) were added to the plates and incubated for 1 hour at room temperature. After another wash step, plates were incubated with 0.05 µg/mL of anti-human, HRP-conjugated secondary antibody (Jackson ImmunoResearch, West Grove, PA). Reactivity was assessed by using a substrate reagent kit to detect HRP (R&D Systems, Minneapolis, MN). To stop the reaction, 2N of H_2_SO_4_ was added to the plate. Absorbance was read on a Multiskan Ascent microplate spectrophotometer (ThermoFisher, Waltham, MA) at 450 nm. Samples with capsid-specific absorbance greater than three times the absorbance of the sample binding to BSA were considered positive. All samples were tested in at least triplicate and experiments repeated at least twice. End point titration was performed on a subset of TAstV-2 positive samples (n = 12).

### Competition ELISA

To monitor the specificity of the assay, a subset of the sera were pre-incubated with 150 µg/mL of recombinant HAstV-1 or TAstV-2 capsid protein or a 1∶4 dilution of chicken astrovirus viral stock (a generous gift of Dr. Holly Sellers, University of Georgia, referenced in [24]) overnight at 4°C prior to use in the ELISA as described above.

### Statistical Analyses

Statistical analyses included Pearson’s Chi square and Fisher’s exact tests to compare categorical variables and unpaired Student *t* tests to compare continuous variables. Odds ratios and associated confidence intervals (CIs) were calculated by using the traditional method. Fisher’s exact method was used if data were sparse. Analyses were performed using SPSS 18.0 software (IBM, Armonk, NY).

## Results

Although there is increasing evidence that astroviruses cross species barriers, no studies to date have examined the seroprevalence of animal or avian astroviruses in humans. Thus, we analysed archived human sera from three cohorts for the presence of turkey astrovirus-2 (TAstV-2) and human astrovirus type 1 (HAstV-1) antibodies by antibody capture ELISA using purified recombinant capsid proteins. Samples with absorbance greater than 3-fold above background (binding to BSA) were considered positive. As expected ∼75% of the people had antibodies against HAstV-1 ranging from 20% in Cohort A to 80–84% in Cohorts B and C (Table 1). It is unclear if the low HAstV-1 positivity seen in Cohort A was due to the young age of some of the participants. Surprisingly 20% of the samples were TAstV-2 positive with the highest number found in Cohort C (26.3%). End-point titration was performed on twelve of the TAstV-2 positive samples and demonstrated that 8% were positive at 1∶1000, 75% were positive to 1∶10,000, and ∼17% were positive to 1∶100,000 dilutions (Table 1). Unfortunately attempts to complement these findings with western blot analysis were unsuccessful and there is not an *in vitro* culture system for TAstV-2 to perform neutralization studies. However, these studies suggest that antibodies against TAstV-2 can be detected in humans.

**Table 1:** Serologic results of TAstV-2 and HAstV-1 antibody testing.

	TAstV-2 positive	HAstV-1 positive
Cohort A (n = 25)	0 (0.0%)	5 (20.0%)
Cohort B (n = 45)	4 (8.9%)^1^	38 (84.4%)
Cohort C (n = 160)	42 (26.3%)^2^	129 (80.6%)

1 4/4 samples positive to 1∶100 dilution.

2 Of 12 TAstV-2 positive samples tested, 8.3% positive to 1∶1000, 75% positive to 1∶10000, and 16.7% positive to 1∶100000.

**Figure 1:**
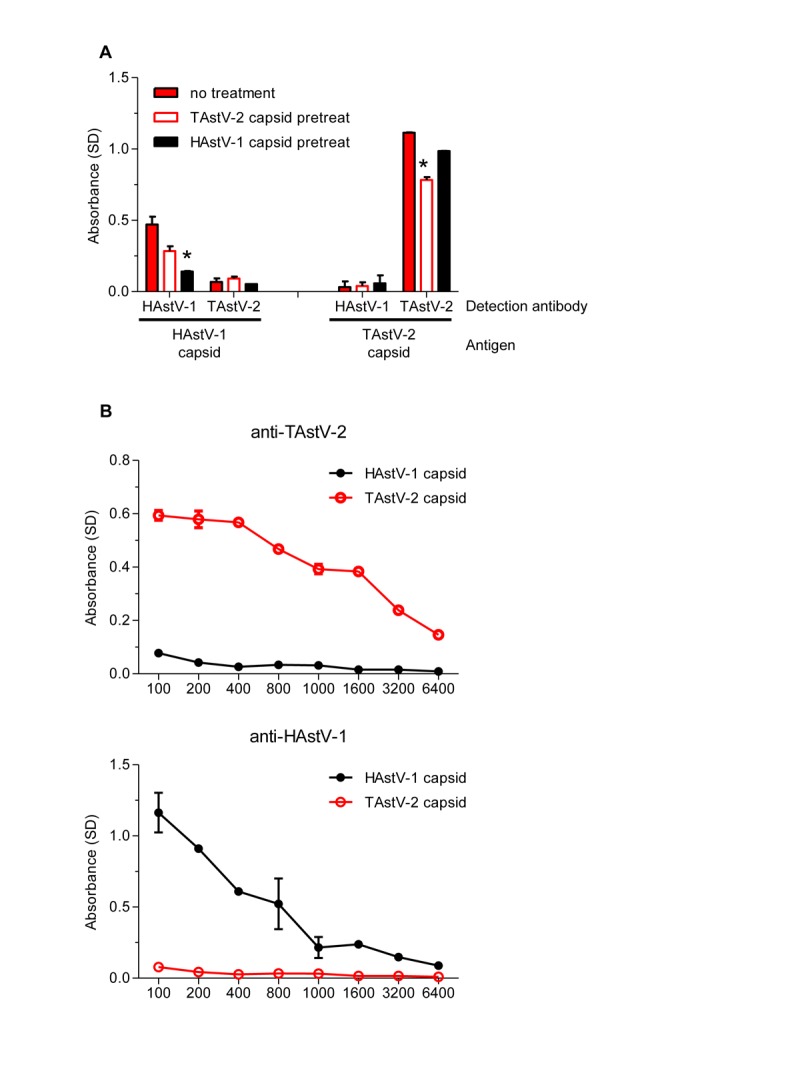
Development of a competitive astrovirus ELISA. A) Rabbit anti-HAstV-1 or TAstV-2 polyclonal antibodies were pre-incubated with PBS (no treatment) or purified recombinant HAstV-1 or TAstV-2 capsid proteins then tested for binding to bound HAstV-1 or TAstV-2 capsid protein by ELISA. *p<0.05. B) Specificity of the rabbit polyclonal antibodies. Samples were run in at least duplicate and data are shown as mean values. Error bars indicate SD.

To ensure that the responses were specific, competition ELISAs were performed. Briefly, random sera samples from all cohorts were pre-incubated with purified recombinant HAstV-1 or TAstV-2 capsid prior to using in the ELISA. If the serum antibodies are specific, pre-incubation with that capsid should competitively inhibit binding to capsid coated on the plate resulting in decreased absorbance. To determine the level of inhibition achievable, polyclonal antibodies produced to specific peptides within the HAstV-1 and TAstV-2 capsids were initially tested (Figure 1A). Pre-incubation of sera with capsid proteins resulted in significant decreases in absorbance. Incubating HAstV-1 capsid with anti-HAstV-1 antibody decreased absorbance by 70.1% (p = 0.0137). In contrast, pre-incubation with TAstV-2 capsid had no significant inhibition. A similar trend was observed with the TAstV-2 antibody where pre-incubation with the TAstV-2 capsid decreased absorbance by 29.7% (p = 0.0018) while the HAstV-1 capsid had no significant effect. Figure 1B demonstrates that the polyclonal antisera are specific to their respective capsid proteins with no cross-reactivity.

**Figure 2:**
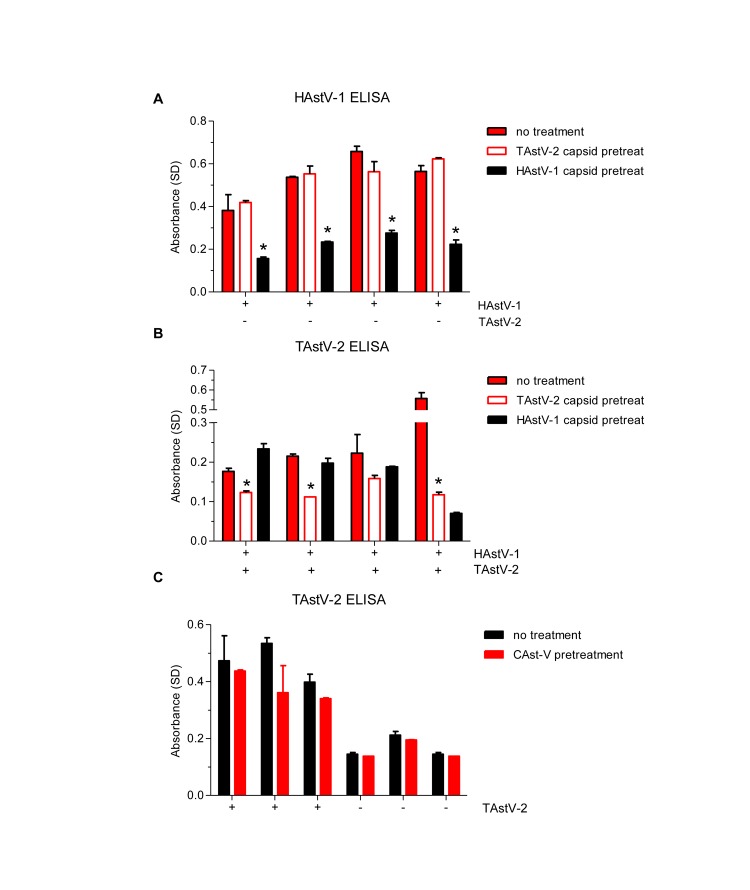
Specificity of the human sera to HAstV-1 and TAstV-2 capsid proteins. A) HAstV-1 positive sera were pre-incubated with PBS (no treatment) or HAstV-1 or TAstV-2 capsid proteins and binding to HAstV-1 capsid protein assessed by ELISA. B) TAstV-2 positive sera were pre-incubated with PBS (no treatment) or HAstV-1 or TAstV-2 capsid proteins and binding to TAstV-2 capsid protein assessed by ELISA. C) TAstV-2 positive and negative sera were pre-incubated with PBS (no treatment) or chicken astrovirus and binding to TAstV-2 capsid protein assessed by ELISA. A plus sign denotes serum sample was positive for indicated virus, and a minus sign denotes serum sample was negative for the indicated virus. Samples were tested in duplicate and data shown are mean values; error bars indicate SD. *p<0.05.

Due to limited quantities, only a small number of human sera could be tested in the competition ELISAs. Of the HAstV-1 positive samples tested, pre-incubating the sera with HAstV-1 capsid led to a significant decrease in absorbance versus human capsid protein (p = 0.0495, 0.0001, 0.0025, 0.0050, respectively). In contrast, pre-incubation with TAstV-2 capsid protein had no effect suggesting that the antibodies we are detecting are specific for HAstV-1 (Figure 2A).

When the converse experiments were performed on TAstV-2 coated plates, pre-incubation of TAstV-2 capsid with the TAstV-2 positive sera led to significant decreases in absorbance (p = 0.0134, 0.0015, 0.0024, respectively) (Figure 2B). Pre-incubation with HAstV-1 capsid did decrease the absorbance of one of the TAstV-2 positive samples (Figure 2B). Finally, to determine if the TAstV-2 positive sera could be inhibited with other avian astroviruses, we obtained a chicken astrovirus (CAstV, [23]) and pre-incubated it with TAstV-2 positive and negative sera and measured binding to TAstV-2 capsid. Incubation with CAstV had no impact on absorbance (Figure 2C). In summation, these studies suggest that the ELISA is measuring HAstV-1- and TAstV-2-specific antibodies.

Twenty six percent of Cohort C participants were TAstV-2 positive. This cohort is composed of a population sampling of adults occupationally exposed to turkeys in the Midwestern US (Iowa and Illinois). In the original cross-sectional study conducted from March 2007 to April 2008, a total of 57 turkey growers, 38 turkey meat processing plant workers, and 82 occupationally unexposed controls from Iowa and Illinois were enrolled after written informed consent was obtained. The demographic characteristics of the study participants have been published [14]. Briefly, most turkey growers used tobacco, were men, and were significantly older than those of other groups. Most growers were white, and most processing plant workers were Hispanic. There were no significant differences in the chronic conditions reported among the groups [14]. “Controls” were study participants who did not interact with turkeys on a daily basis at the workplace (i.e., those who were not turkey growers or meat-processing plant workers) [14].

The majority of the study subjects (80.6%) were HAstV-1 positive and there was no difference in positivity between the study groups. In contrast, we detected significant differences between the study groups testing TAstV-2 positive. Antibodies were detected in 21.1% of the non-occupationally exposed subjects, 21.6% of the turkey growers, and 45.5% of the processing plant workers (p = 0.019) (Table 2). The odds of testing positive for TAstV-2 antibodies among processing plant workers was about 3 times more than that among turkey growers or non-occupationally exposed participants (OR = 3.1, 95% CI: 1.3–7.5). Turkey growers did not statistically differ from non-occupationally exposed participants (OR = 1.0, 95% CI: 0.4–2.5).

**Table 2:** Serologic and demographic results of Cohort C.

Antibody Presence	Occupationally unexposedgroup, no. (%) (*N* = 76)	Meat processing plantworkers, no. (%)(*N* = 33)	Turkey growers,no. (%) (*N* = 51)	*P* value^1^
TAstV-2 antibody				
Positive	16 (21.1%)	15 (45.5%)	11 (21.6%)	0.019
Negative	60 (78.9%)	18 (54.5%)	40 (78.4%)	
HAstV-1 antibody				
Positive	60 (78.9%)	29 (87.9%)	40 (78.4%)	>0.05
Negative	16 (21.1%)	4 (12.1%)	11 (21.6%)	

1 p-value associated with pearson Chi-square test.

We then focused on determining the risk factors that may be associated with having TAstV-2 antibodies among the processing plant workers. Several potential risk factors were examined including age, sex, race and ethnicity, smoking, and chronic diseases. A set of occupational risk factors such as the use of gloves, masks, aprons, boots, and eye protection along with the specific tasks the individual performed in the plant were also studied. None of these variables were significantly associated with having TAstV-2 antibodies.

## Discussion

Nothing is known about the seroprevalence of non-human mammalian and avian astroviruses in humans. Our results suggest that a minority of people have antibodies to TAstV-2; however, occupational exposure to turkeys can result in seroconversion. This risk increases by working in a meat processing plant, but not by raising turkeys. Processing plant workers spend more time in contact with turkeys than do turkey growers, which may explain the decreased evidence of seroconversion among turkey growers. Processing plant workers have a wide spectrum of activities within the meat processing plant, with exposure to live birds occurring when unloading turkeys from transport vehicles, handling birds within the plant, and cleaning and decontaminating trucks and cages where live birds have been contained. These workers are also routinely exposed to bird carcasses and body fluids. In our study, workers involved in slaughtering the birds did not have a significantly higher risk of seroconversion than other workers did. In fact, none of the measured risk factors was associated with an elevated antibody titer against TAstV-2. This outcome could be due to the small sample size of processing plant workers.

Why did the “occupationally unexposed” group in Cohort C have TAstV-2 antibodies? One possibility is cross-reactivity of the HAstV-1 and TAstV-2 antibodies. The HAstV-1 and TAstV-2 are genetically and structurally distinct [8], [23] and antibodies produced to the specific capsids do not cross-react. Competitive ELISAs demonstrated TAstV-2 positive sera were inhibited by pre-incubation with TAstV-2 capsid, although inhibition may not have been complete. This was not unexpected given that we were unable to completely inhibit rabbit anti-TAstV-2 antibody binding by pre-incubation with recombinant capsid protein. An alternative possibility is exposure to a different avian astrovirus that cross-reacts with the TAstV-2 antibody. Several strains of TAstV as well as chicken astroviruses (CAstV) are known to circulate in commercial poultry in the United States [25]. Although we cannot definitively rule out this possibility, we found that CAstV failed to inhibit TAstV-2 responses when pre-incubated with TAstV-2 positive human sera. However, one caveat to this might be that CAstV viral stocks were used for this assay, and the concentration of CAstV capsid was probably far lower than a purified recombinant capsid stock. Based on these findings, and given that our two additional cohorts had no-to-minimal TAstV-2 positivity, we would hypothesize that although not occupationally exposed to turkeys, this population, which is based in rural Iowa and Illinois, was unknowingly exposed to TAstV-2. One possibility is environmental exposure. Astroviruses are extremely stable and can persist in the environment for long periods on surfaces [26]. Thus, employees in a turkey processing plant may be exposed to TAstV-2 virus without direct contact with turkeys. Environmental testing of abattoirs for TAstV-2 could prove interesting.

In conclusion, our results suggest that occupational exposure to turkeys in processing plants is a risk factor for exposure to TAstV-2. Is this finding unique to TAstV-2? We won’t be able to answer this question until reagents to additional avian and non-human mammalian astroviruses become available. Future studies are also needed to determine whether such seroconversion events are actually associated with clinical disease.
